# Supplementary material for: Innovative approach for potential scale-up to jump-start simplified management of sick young infants with possible serious bacterial infection when a referral is not feasible: Findings from implementation research
Source: PLoS One. 2021 Feb 5;16(2):e0244192. doi: 10.1371/journal.pone.0244192 (PMC7864440; doi:10.1371/journal.pone.0244192)
Supplement: Attachment — Submitted filename: plos one review.docx [file pone.0244192.s001.docx]

Abstract

Title is very long, considering streamlining

What do you mean by “Sick” – please define

What is PSBI – not defined in abstract (I see it is defined later in introduction)

Define critical illness, clinical severe illness, fast breathing pneumonia (not typical phrases used)

Initially says children aged <2 months and then say 7-59 days – need to be consistent

Introduction

*An estimated 2.5 million neonatal deaths occurred in 2018 contributing to 47% of under-5 deaths [1].* – where is this? Worldwide? Numbers specific to Ethiopia would be more appropriate

*Neonatal infections including pneumonia, sepsis and meningitis are one of the major causes resulting in nearly 55000 deaths* – sepsis is a clinical diagnosis from a variety of infections (including pneumonia and meningitis), more appropriate here would be bacteremia

*In 2011, the Government of Ethiopia trained HEWs to manage sick young infants with signs of PSBI with oral amoxicillin plus gentamicin for seven days when referral was not feasible, but the utilization of this intervention was low* – what were the specific barriers identified? You note the use of oral/IM antibiotics is low, but you should specify the current rate.

*Thus, the objective of this implementation research was to implement a simplified antibiotic regimen (2 days gentamicin injection and 7 days oral amoxicillin)* – you state the standard of care is gentamicin and amoxicillin for 7 days, what is the evidence that this decreased length of treatment of gentamicin is acceptable?

Study Population

*unable to feed at all or not feeding well* – This can be called “poor feeding”

*no movement at all or movement only when stimulated* – This can be called “listlessness” or “lethargy”

Are the clinical signs of PSBI what you are referring to as “sick” young infants – this needs to be clarified

Intervention

The information about the health system should be under context and not intervention.

The rest of the intervention section about training HEWs should be part of the introduction along with your initial statement of objective. You have an intervention section and then implementation section that should be combined as one. Can have subsections on “Planning the intervention” and then “interventions”

assessed and classified them for PSBI – what does this entail? Classification not described.

Please define which infants were recommended for referral

Why are families refusing referral? This barrier needs to be described in more detail

Primary outcome

Your objective is not framed as a SMART aim. What was the current rate of identification and treatment of infants with PSBI and in what time frame is this trying to be accomplished?

What is your definition of appropriate treatment? The rate of antibiotic adherence is a secondary outcome so this must be different.

Data analysis

There is only “descriptive analysis” – reporting data but without context of any comparison to baseline or trend over time which is not consistent with standard implementation research. This does not allow any conclusions to be drawn as to the effects of the interventions.

Phases of implementation

*A sensitization workshop* – what is this? This is not a typical term (you describe it but I’m not sure why it is called sensitization)

Training of health care pproviders – typo

*Once at the health post, the HEW assessed and classified the sick young infant. If the sick young infant was found to have clinical severe infection or critical illness* - what is this definition?

Fast breathing pneumonia is not a typical term. Do you mean respiratory distress that would indicate a possible pneumonia? How was this identified?

Were there PDSA cycles? Changes made to the interventions/implementation based on real-time data? The description of all stakeholders and educational interventions as well as process changes were thoroughly described, but a timeline of implementation would be helpful to understand what order changes occurred in (or if it was all at once), and what impact this had on the data.

Results

*We estimated that 25% of live births were not identified by HEWs in this study (n=1964)* – why are some not identified and where is this number coming from?

*In the initial stages of implementation, the rate of identification and management of sick young infants with PSBI by HEWs was low. This improved after six months of implementation (Figure 1).* – the rate is not on this figure, it is the total number of cases, so this is not consistent data reporting.

Figure 1 is a bar graph – for quality improvement/implementation research, analysis should be done using a run chart or statistical process control chart (preferred). This would allow statistical significance to be identified. The key interventions contributing to any changes on the chart can be indicated so that correlation can be seen. Data can be broken down into weekly or biweekly so that there are enough data points to make a statistical process control chart.

What is the baseline data pre-implementation? There is no comparison, only description of results after implementation.

Deaths

*deaths occurred among the 144who were referred by HEWs* - typo

Implementation challenges and solutions

This section should be incorporated into the description of the interventions, not after the results. Preceding barriers can be described first in the context of why the interventions were chosen. If applicable, each problem and solution should be grouped with the description of the intervention itself. Ongoing challenges can be described in the discussion in the context of next steps.

It seems much of what is described in this section are the initial barriers identified – this needs to be described earlier in the manuscript as context for the implementation phases.

*This resulted in improvement in the skills to carry out activities as per standard case management in the subsequent quarters*. – where is this data?

The discussion of poorly equipped neonatal intensive care units does not seem relevant to the study.

The TSU had been focusing on referral and this has improved significantly after the first quarter. - Where is the data on referral rates? There is no description of this analysis or figure to demonstrate statistical significance. This cannot be stated unless clearly demonstrated.

Discussion

The discussion needs to relate back to your primary outcomes. Did you achieve your goals?

Data is being compared against different studies and conclusions are being made without data analysis showing statistical significance with the implementation of these interventions.

Was investigating whether this alternate antibiotic treatment was effective an outcome? There is emphasis on the success of this, but not listed as an objective.

*Our study had several strengths.* – This should be phrased as keys to successful intervention implementation

General issues:

-terminology is interchanged and unclear throughout. What is the difference between sick young infants, PSBI, critical illness and clinical severe illness? These terms need to be defined and using distinctly.

-Unclear whether this study is looking at infants <2 months or 7-59 days as both are used

-More sophisticated data analysis should be used. For quality improvement/implementation research, a run chart or statistical process control chart (preferred) is necessary to demonstrate significant change due to interventions.

-There is no statistical significance reported for data analysis. The data is reported without any analysis provided. Overall rates are reported but there is no comparison to baseline data or trends over time. This is not standard for implementation research and prevents any conclusions to be made in terms of effectiveness of the interventions.

-There is no comparison of post-implementation data to a baseline data set. Baseline rates reported as “low” but the data are not reported. This prevents any conclusions that the interventions were successful.

-A key driver diagram is essential to a quality improvement/implementation project. This should be included to demonstrate the issues/barriers and linkage with interventions to achieve aims.

-The discussion of the interventions and practical strategies for implementation is strong. However, the data analysis is not strong enough to support conclusions of success. This manuscript is more descriptive of intervention implementation and barriers/solutions than true quality improvement research.

-There are so many different facets of this project and elements of data described, it may be helpful to narrow the focus so the key outcomes can be more prominent.

-The manuscript is very long. Please streamline where appropriate.

-occasional grammatical issues and typos throughout
