# Supplementary material for: Innovative approach for potential scale-up to jump-start simplified management of sick young infants with possible serious bacterial infection when a referral is not feasible: Findings from implementation research
Source: PLoS One. 2021 Feb 5;16(2):e0244192. doi: 10.1371/journal.pone.0244192 (PMC7864440; doi:10.1371/journal.pone.0244192)
Supplement: Attachment — Submitted filename: Point by point responses_reviewers_28 Aug 2020.docx [file pone.0244192.s002.docx]

**Point by point responses to reviewers’ comments**

**Journal:**PLOS ONE

**Manuscript ID**: PONE-D-08171

**Manuscript Title*:*** *Innovative approach for potential scale-up to jump-start simplified management of sick young infants with possible serious bacterial infection when referral is not feasible: findings from implementation research*

Dear editor and reviewers,

We appreciate the editor and reviewers’ comments which were constructive and genuine. We have made Point by point responses to reviewers’ comments. We have also attached the clean and highlighted versions of the document.

We would like also to appreciate PLOS ONE editor for their support in extending the due date as a result of the challenges we encountered. We believe that our revised manuscript would answer the major issues raised by the reviewers. We are happy to address further comments from the reviewers and editor.

**Reviewer 1**

**Context and setting**

**Comment #1**

Correct discrepancy – Last sentence of introduction states: “In two regions in Ethiopia, Tigray and Jimma” while methods mention only two districts of Tigray (nothing in Jimma). Issue occurs again in last sentence of data management section: “Common data collection tools and the data base were used by both the Tigray and Jimma sites”.

**Authors’ response**

Thank you and we agree with the reviewer’s comment. The discrepancy is corrected. The paper now focuses on the results from Tigray.We have added the following text in the revised manuscript:

“*Thus, the objective of this implementation research was to implement a simplified antibiotic regimen (2 days gentamicin injection and 7 days oral amoxicillin) for management of sick young infants with PSBI in a programsetting when referral was not feasibleto achieve anoverall treatment coverage of at least 80% in two districts in Tigray, Ethiopia*.”

Page 6; line 102 - 105 of the revised manuscript.

**Comment #2**

Would be useful to provide readers a bit more information on characteristics of HEWs (female, education and training levels). In addition, 17 activities of HEWs mentioned but not described- suggest giving examples and / or providing reference for people to have more info on HEWs and their roles.

**Authors’ response**

Thankyou for this good suggestion. However, we believe that by adding all these details, the length of the manuscript will increase substantially. But as suggested by the reviewer, we have provided a reference in the revised manuscript for more detailed background information on the Health Extension Programme in Ethiopia. Please see newly added reference #20. [*Teklehaimanot, H.D., Teklehaimanot, A. Human resource development for a Community-based health extension program: a case study from Ethiopia. Hum Resour Health 2013; 11:39.* [*http://www.human-resources-health.com/content/11/1/39*](http://www.human-resources-health.com/content/11/1/39)].

Page 7; line 125– 126 of the revised manuscript.

**Comment #3**

Health system is well described but there is little information provided on the community (beyond the population size) - recommend to also provide short description of the community socio-demographics.

**Authors’ response**

Thanks. We have added the following information in the revised manuscript:

*The two districts are characterized by lowland and highland agro-ecological conditions. Mixed farming is widely practiced in the two districts. The livelihoods of the two districts mainly depends on seasonal rain fed subsistence agriculture. Moreover, irrigation farming is used in the low lands of both districts to complement rain fed agriculture.* *The total population of the two study districts was 260,884 (*reference 17*)****.***

Page 6; line 109–113 of the revised manuscript.

**Study population**

**Comment #4**

The study tracked all births during the study period to determine the denominator for estimated coverage – therefore the study population should be expanded to indicate that all babies born during the study period were included in the study and differentiate between those infants that were identified as sick and then treated through the study.

**Authors’ response**

We agree with the reviewer and we have made the necessary modification. All babies born during the study period were included in the revised manuscript.

*All babies born during the study period were included in the study.*

Page 7; line 141 of the revised manuscript.

**Primary and secondary outcomes**

**Comment #5**

These are not sufficiently described or defined. Each primary and secondary outcome should be defined, ideally in a table. In addition, secondary outcomes should be expanded to include other indicators that are reported on in the results (e.g. follow up, accuracy of HEWs)

**Authors’ response**

Thanks for pointing out this issue. We have modified the revised manuscript as follows:

*‘The primary outcome target was at population-level identification and treatment of young infants aged up to 2 months with any sign of PSBI of at least 80% (coverage); and 80% identified young infants with any sign of PSBI who receive appropriate treatment. Appropriate treatment was defined as the provision of inpatient treatment (injectable gentamicin and ampicillin or any other appropriate antibiotics) or outpatient treatment (oral amoxicillin twice daily for at least 5 days plus IM gentamicin injection for 2 days) to sick young infants with PSBI when referral was not feasible.’*

S**econdary outcomes**

*Secondary outcomes included infant death rate within two weeks after the initiation of inpatient or outpatient treatment; clinical deterioration defined as emergence of any sign of critical illness or new sign of clinical severe infectionduring the outpatient treatment ofsick young infants; any serious adverse effects such as swelling local to injection site, new onset of rash, disseminated and severe rash, anaphylactic reaction, stopped passing urine for >12 hours (renal failure) and cellulitis or abscess at injection site in young infants who received simplified antibiotic treatment at health post level, rate of adherence to outpatient treatment and accuracy of classification of PSBI by HEWs*.

Page 7 - 8; line 145 - 150

**Sample size determination**

**Comment #6**

Sample size determination is missing – Should be added to methods section

**Authors’ response**

Thanks. A section on sample size determination is included as per the suggestion from the reviewer.

*The sample size was determined based on 3.1% crude birth rate per year in Ethiopia (reference 18 - DHS Ethiopia, 2016). The population of both districts was 260884 (reference 17). Thus, it was calculated that there would be 8087 births per annum in both districts. Assuming 10% incidence rate of PSBI in young infants <2 months old (references 11-13), a sample size of 809 young infants with any sign of PSBI in the first 2 months of life was required to show the 80% coverage.*

Page 7; line 136– 139 of the revised manuscript.

**Intervention**

**Comment #7**

This section repeats information on the health system covered on content and that could be removed. Information on follow up schedule (when, where, by who) should be included here.

**Authors’ response**

Thanks. Redundant information on health system in the intervention section is removed.

Information on follow up was added as follows:

Page 12; Line 257 - 259 of the revised manuscript.

**Data management**

**Comment #8**

should be revised to “Data collection and data management” and include information on the schedule for data collection (day 3, 7? Etc.)

The reviewer is not asking for explanation but is asking to change the title of the section and then to include info on schedule for data collection

**Authors’ response**

Thanks. We have revised the title and included information aboutdata collection as suggested by the reviewer.

*The sick young infants with PSBI were identified by HEWs either during the home visits or health facility visits.* *Cases were also identified during routine postnatal home visits made by HEWs to promote essential newborn care in the first six weeks (on days 1, 3, 7 and 42) of life.**All young infants who are provided treatment were evaluated daily by the same treating HEW and by cluster supervisors on day 5 and day 8 for the ascertainment of treatment outcome (improvement or deterioration).*

Pages12; line 254– 259 of the revised manuscript.

**Implementation Phases**

**Comment #9**

This section is very long and while important, could be condensed using tables with additional information provided in supplementary files. Specifically- recommend covering roles and responsibilities of each stake holder group in a table (either in paper or supplementary file).

**Authors’ response**

Thanks. The roles and responsibilities are removed from the main body of the document and have been included as a table 1.

Pages 28; line 581, Table 1. Roles and responsibilities of the various stakeholders.

**Comment #10**

It would help the reader to include a figure showing how cases of sick young infants were identified, referred, treated and followed up.

**Authors’ response**

Thanks. Figure 2 representing the schematic representation of the identification, classification, referral, treatment and follow up of the sick young infants has now been included in the result section of the revised manuscript.

Figure2: Schematic representation of the identification, assessment, classification, referral, treatment and follow up of the sick young infants.

**Comment #11**

Some content under patient identification should be included in results or discussion section-specifically: “In the initial stages, the demand side was weak which improved, particularly in those areas where WDGs and their leaders were more active. It resulted in better utilization of CBNC and PSBI services and building trust and confidence in the HEWs and health posts to treat and manage newborn illnesses at the community level when referral was not feasible”

**Authors’ response**

Thanks. The section specifically: “In the initial stages, the demand side was weak which improved, particularly in those areas where WDGs and their leaders were more active. It resulted in better utilization of CBNC and PSBI services and building trust and confidence in the HEWs and health posts to treat and manage newborn illnesses at the community level when referral was not feasible” is moved to the discussion section and modified to:

As a complementary strategy to improving PNC home visits, reinforcing and strengthening the links between the health extension program and the community through the active participation of the WDGs (women development groups) and the links with the midwives who support the skilled deliveries of mothers in the health facilities (health centers/hospitals) is highly recommended.

Page17-18; line 374 – 378.

**Comment #12**

Incentives- Information on incentives to HEWs is included under the “Roll of the District Health Office” but should be included under the “Implementation Phase” with its own sub heading. Any other incentive provided through the study should be described here as well.

**Authors’ response**

Thanks. A sub heading is included under the implementation phase. The HEWs from the health posts were given around US$ 2 for completing each CRF and the clinical mentor was given US$ 200 monthly to compensate for his/her time.

Page 12 line 246 - 248

**Results**

**Comment #13**

A table with results for the 117 children that accepted referral to the hospital and the 333 children who went directly to the hospital should be included (if not possible should be explained why not)

**Authors’ response**

Thank you for this comment. We only had the resources to actively follow those young infants who were treated who were treated on an outpatient basis at the health posts by HEWs or at the health center level, both part of the primary health care unit. We did collect the outcome data for those who referred from either a health post or the health center (117 clinical severe infection and 27 critical illness) on day 14 whether they were alive or dead (7 died and 137 recovered). However, we didn’t have the resources to follow actively 333 children who went directly to the hospital as they never presented to any of the health posts or a health center in the study area. We collected their data *post hoc* from the catchment area hospitals to calculate the overall coverage of treatment for the population of young infants. Further information could not be collected for the children treated at the hospitals.

**Comment #14**

Implementation challenges and solutions- A long list is provided, and it would be helpful for the reader to categorize or group these challenges somehow (perhaps into what stage-patient identification, referral, treatment follow up)

**Authors’ response**

Thanks. The table is modified and made to include a column on the stages of patient identification, referral, treatment and follow up.

Page 33; line 593.**Table 4. Implementation challenges encountered and solutions provided.**

**Discussion**

**Comment #15**

First sentence should include reference to identify the studies referred to

**Authors’ response**

Thanks. The section is re-written again.

Page 15 - 17; line 324 - 354

**Comment #16**

What are the reasons that acceptance of referral was so high in this setting? This contrasts many other studies and deserve some attention in the discussion - particularly given the mortality that occurred there.

**Authors’ response**

Thanks.We believe the main reasons for the better utilization of services and higher referral acceptance was most probably the presence of relatively activeWDGs and their leaders to some extent especially after the initial implementation phase, free ambulance service and free treatment of neonates at the receiving health facilities.Itresulted in a better utilization of CBNC and PSBI services and building trust and confidence in the HEWs and health posts to treat and manage newborn illnesses at the community level when referral was not feasible

Page 16; line 331 - 335

**Comment #17**

The sentence “To effectively implement PSBI case management ,the first step is to track all pregnant women and births” is misleading – It is possible to provide PSBI case management without this (as per Malawi example), but facility delivery levels and care seeking must be high – so should be reworded to be specific to this context.

**Authors’ response**

Thanks. Yes, it is possible to provide PSBI case management through high facility delivery and care seeking and without tracking all pregnant women and births. But, in our setting, we would rather argue that tracking all pregnant women and births would be essential to effectively implement PSBI case management at the community level (health post level). Unless, pregnancies and births are tracked, it is very difficult to identify sick young infants with PSBI and also conduct PNC visits since delivery services are not provided in the Health Posts by the HEWs. Pregnant women tracked by the HEWs are referred to the Health Centers or Hospitals for facility delivery by the HEWs. Such mothers and their newborns can easily be linked to the HEWs and hence can make all the necessary postpartum follow ups. However, the possibility of linking the mother and the newborn immediately after delivery to the HEWs would be minimum if the pregnant woman is not tracked by the HEW or known to the HEW particularly in the rural communities similar to our study communities. HEWs will be unable to track all live-births which will result in low detection rates of possible serious bacterial infection in young infants.

Thus, we would argue for the message to remain as it is but we modified the sentence to address the reviewer concern and have re-written it as follows:

To effectively implement PSBI case management, an important step is to track all pregnant facilitated postnatal home visits, especially in the later part of the implementation. The strategy of WDGs was conceptualized as a way to identify pregnancies and births, create demand for health care, wellness, and improve access to health care

Page 17; line 355 - 356

**Comment #18**

Study recommends strengthening postnatal home visits – What specifically should be done and how effective are these likely to be. The literature shows that obtaining high coverage of PNC home visits has not been successful in most settings (<20% coverage) – suggest referring to analysis by McPherson

<https://www.ncbi.nlm.nih.gov/pmc/articles/PMC6005634/>) and providing potential alternative/complementary strategies to increase identification of sick young infants

**Authors’ response**

Thanks. It is true that obtaining high coverage of PNC home visits is a challenge even in the study communities. However, we still argue that strengthening the PNC home visits is key to the uptake of PSBI services at the community level when referral is not feasible, of course.

Timely provision of postnatal care services and prompt identification and appropriate care-seeking for sick young infants helps in reducing neonatal mortality^[1]^ [reference 27]. Large numbers of women and their newborns remain at home during and immediately after birth, so building and reinforcing the links between the community and health facilities is essential to improve the provision of postnatal services. Strengthening this link will be key for the successful implementation of PSBI case management at a community level in the Ethiopian setting. Thus, as a complementary strategy to improving PNC home visits, reinforcing and strengthening the links between the health extension program and the community through the active participation of the WDGs (women development groups) and the links with the midwives who support the skilled deliveries of mothers in the health facilities (health centers/hospitals) is highly recommended.

Page 17; line 374 - 378

**Comment #19**

High turnover of HEWs is mentioned as a limitation only in passing but would be quite important to understand – this should be elaborated on (What level of turnover, what possible reasons and what might be done to limit it)

**Authors’ response**

Thanks. As indicated by the reviewer, it is quite important to understand the reasons of high turnover.

The high turnover of the HEWs is another limitation, which are the result of high workload, absence of career structure in the health sector for HEWs and lack of incentives. The situation was felt during the monthly field visits as we were meeting new faces of HEWs. In order to address the high turnover of the HEWS, increasing the number of HEWs per health post (at least 3), creating career structure for HEWs and the construction of residential houses within the premises of the health posts were discussed with the health authorities to limit the fast turnover.Nonetheless, findings are likely representative of patterns of PSBI case management in rural Ethiopia.

Page 20; line 426 - 431

**Comment #20**

Discussion should address how generalizable are the findings to other settings in Ethiopia and how likely these results are to be sustained in the absence of the study support and incentives.

**Authors’ response**

Thanks. The suggestion from reviewer is well taken and the discussion section is modified accordingly

*Our findings are sustainable in the absence of the study support and incentives because the implementation was entirely done by the existing health system with modest refresher trainings. These refresher trainings can easily be conducted by the health work force from the nearby health facilities (health centers and hospitals). Moreover, they are generalizable to the Ethiopian setting because the study communities are similar and the CBNC programs are implemented following the same strategies. Besides the study tools (CRFs) were adapted from the CBNC register which is used by HPs throughout the country.*

Page 19; line 401 - 407

**Comment #21**

Minor comments: Paper should receive a careful final edit as there are several typos and incomplete sentences.

**Authors’ response**

Thanks. The typos have been addressed and the revised manuscript was reviewed by a native English speaker.

**Reviewer 2**

**Introduction**

***Comment #1***

*An estimated 2.5 million neonatal deaths occurred in 2018 contributing to 47% of under-5 deaths [1].* – Where is this? Worldwide? Numbers specific to Ethiopia would be more appropriate

**Authors’ response**

Thanks. Yes, it is worldwide. As suggested by the reviewer we have modified the statement to include information from sub-Saharan Africa (SSA), as we could not find Ethiopia-specific information. We believe that the data for Ethiopia is similar to that of SSA.

*An estimated 2.5 million worldwide and 1 million neonatal death in Sub Saharan Africa occurred in 2018 contributing to 36% and 47% of under-5 deaths, respectively.*

Page 5; line 75 - 76

**Comment #2**

Neonatal *infections including pneumonia, sepsis and meningitis are one of the major causes resulting in nearly 55000 deaths* – sepsis is a clinical diagnosis from a variety of infections (including pneumonia and meningitis), more appropriate here would be bacteremia.

**Authors’ response**

Thanks. Sepsis could be from a variety of causes. We have modified the sentence a bit. Neonatal infections primarily bacterial in origin, including pneumonia, sepsis and meningitis are one of the major causes of 55000 neonatal deaths every year

Page 5; line 76 - 78

**Comment #3**

The government of Ethiopia trained HEWs to implement community based newborn care which includes PSBI management when referral was not feasible in selected communities between 2008 - 2013, but *the utilization of this intervention was low* – what were the specific barriers identified? You note the use of oral/IM antibiotics is low, but you should specify the current rate.

**Authors’ response**

Thanks. Comment is well taken and modified as follows.

The Government of Ethiopia trained HEWs to implement community based newborn care which included PSBI management when referral was not feasible in selected communities in three regions between 2008 - 2013, but the estimated treatment coverage was only around 50%. [15]. Some of the reasons for this low coverage were lack of awareness, perceived illness severity, perceived early treatment by mothers and having other young children [16].

Page 6; line 97 - 102

**Comment#4**

*Thus, the objective of this implementation research was to implement a simplified antibiotic regimen (2 days gentamicin injection and 7 days oral amoxicillin)* – you state the standard of care is gentamicin and amoxicillin for 7 days, what is the evidence that this decreased length of treatment of gentamicin is acceptable?

**Authors’ response**

Reviewer has raised an important point. We have outlined this in the introduction of the manuscript, but will briefly summarize here again. In low resource settings most families who were referred for hospitalization and inpatient treatment for clinical sepsis refused referral advice and opted for treatment near to their homes [references 7-9]. Trials were conducted in Africa and Asia to address this issue, which compared various simplified antibiotic regimens with a reference regimen of injectable gentamicin and injectable penicillin for 7 days [references 10-13]. Data form these trials showed that simplified regimens were as effective as the 7-day course of injectable antibiotics for some sick infants who had signs of ‘clinical severe infection’, a sub-category of PSBI. The two days injectable gentamicin and 7 days of oral amoxicillin were equally effective as the 7 days gentamicin and 7 days oral amoxicillin where referral was not feasible [11].The simplest regimen, which included only two injections and oral antibiotic treatment, had both the lowest treatment failure rate and the highest adherence to treatment [11].The evidence was used by WHO to develop a guideline for managing young infants with PSBI when referral was not feasible [reference 14], which recommended both 2 days and 7 days of gentamicin plus 7 days of oral amoxicillin for management of clinical severe infection.

Page 5: line 90 - 93

**Study population**

**Comment #5**

Unable to feed at all or not feeding well – This can be called “poor feeding”

no movement at all or movement only when stimulated – This can be called “listlessness” or “lethargy”

Are the clinical signs of PSBI what you are referring to as “sick” young infants – this needs to be clarified

**Authors’ response**

The reviewer is right that together the feeding problems can be called ‘poor feeding’ and movement problems can be called ‘listlessness or lethargy’. We have used terminologies that have been developed by WHO to identify sick young infants with signs of PSBI and to further differentiate them when referral is not feasible into those with signs of ‘clinical severe infection’ (a sub-category of PSBI) to be treated with simplified antibiotic regimens on an outpatient basis and those with ‘critical illness’ who can be best treated only in a hospital [references 14 and WHO Integrated Management of Childhood Illness chart booklet^[2]^]. ‘Unable to feed at all’ and ‘no movement at all’ are signs of critical illness and ‘not feeding well or stopped feeding well’ and ‘movement only when stimulated’ are signs of clinical severe infection. A list of signs of critical illness and severe clinical infection are as follows. Critical illness - convulsions, unable to feed at all, no movement at all and clinical severe infection fast breathing [60 breaths per minute or more] in a young infant age less than 7 days, severe chest indrawing, high body axillary temperature [38.0^o^C or above] or low body axillary temperature [less than 35.5^o^C], not feeding well and movement only when stimulated.

These definitions are given under Panel 1

Page 26, line 577.*Panel 1.Definitions and management strategies of young infants with signs of PSBI – adapted by Government of Ethiopia from WHO Guideline [14]*

**Intervention**

**Comment #6**

The information about the health system should be under context and not intervention.

**Authors’ response**

Thanks. We have moved it from intervention to the context section.

The health system structure in Ethiopia is a three-tiered health system structure of primary care (primary health care unit), secondary care (general hospital) and tertiary care (referral hospital). The Primary Health Care Unit (PHCU) at the bottom level consists of health posts that serve 5000, health centers that serve 25000 and a primary hospital which serves 100000 people. The PHCU feeds into the secondary level of health care, i.e. general hospital, which serves a population of one million and the general hospital in turn feeds in to the referral hospital which serves about 5 million people.

Page 6 - 7; line 119 - 125

**Comment#7**

The rest of the intervention section about training HEWs should be part of the introduction along with your initial statement of objective

**Authors’ response**

Thanks. We believe that the HEW part of this section would not fit well in the introduction of the paper because it was one of the activities conducted during the implementation research.

## Page 9; line 184: Training of health care providers

**Comment#8**

You have an intervention section and then implementation section that should be combined as one. Can have subsections on “Planning the intervention” and then “interventions”

**Authors’ response**

Thanks. The two sections were merged and renamed as per reviewer’s suggestion.

Page 8: line 160, planning the intervention

Page 10:line 196, Intervention

**Comment #9**

Assessed and classified them for PSBI – what does this entail? Classification not described.

**Authors’ response**

Thanks. The procedure involved identification, assessment, classification and treatment based on the WHO Guideline [14]. We have explained this in a panel (see panel 1)

Page 26, line 577.*Panel 1.Definitions and management strategies of young infants with signs of PSBI – adapted by Government of Ethiopia from WHO Guideline [14]*

**Comment #10**

Why are families refusing referral? This barrier needs to be described in more detail

**Authors’ response**

Thanks. *The barriers for referral refusal are long travel distance, low health seeking behavior and cultural barriers as postpartum mothers confined themselves in their homes till the time of baptism (40 and 80 days for boys and girls after delivery respectively).*

We have described this on

Page 16; line 337 - 344

**Primary outcome**

**Comment#10**

Your objective is not framed as a SMART aim. What was the current rate of identification and treatment of infants with PSBI and in what time frame is this trying to be accomplished?

**Authors’ response**

Thanks. Comment is well taken. We have revised the primary objective on page 6 and the primary outcome on page 8 in light of reviewer’s comments. Also see responses to Reviewer 1 comments 1 and 5.

**Comment #11**

What is your definition of appropriate treatment? The rate of antibiotic adherence is a secondary outcome so this must be different.

**Authors’ response**

Thanks. Appropriate treatment was defined as the provision of inpatient treatment (injectable gentamicin and ampicillin or any other appropriate antibiotics) or outpatient treatment (oral amoxicillin twice daily for at least 5 days plus IM gentamicin injection for 2 days) to sick young infants with PSBI when referral was not feasible.

Page7 - 8; line 147 - 150

**Data analysis**

**Comment#12**

There is only “descriptive analysis” – reporting data but without context of any comparison to baseline or trend over time which is not consistent with standard implementation research. This does not allow any conclusions to be drawn as to the effects of the interventions.

**Authors’ response**

Thanks. Yes, we have used a descriptive analysis. But we believe that our descriptive analysis is robust enough to support our results. We have mentioned that before this implementation research the coverage of treatment for sick young infants with signs of PSBI was around 50%.

Quantitative data were cleaned and entered into SPSS version 20 software. After checking for missing data, outliers and invalid values, descriptive analysis was conducted to report frequencies, coverage and proportion of sick young infants with clinical severe infection, critical illness, pneumonia, local bacterial infections; treatment failures; referrals and treatment outcomes along with relevant information regarding PSBI management and treatment at the community level.

Page 13; line 261 – 266

**Phases of implementation**

**Comment # 13**

*A sensitization workshop* – what is this? This is not a typical term (you describe it but I’m not sure why it is called sensitization)

**Authors’ response**

Thanks. We have renamed it a sensitization meeting, in which a briefing was given to the stakeholders in the study communities to sensitize them about the intervention to increase coverage of treatment for sick young infants with signs of PSBI and to create awareness about the and the implementation research.

Page 9; line 176 Sensitization Meetings

**Comment#14**

Training of health care providers – typo

**Authors’ response**

Thanks. Addressed as per the comments.

Page 9; line 184. Training of health care providers

**Comment #15**

*Once at the health post, the HEW assessed and classified the sick young infant. If the sick young infant was found to have clinical severe infection or critical illness* - what is this definition?

**Authors’ response**

Thanks. This is defined in the study population section.

Critical illness includes no movement at all,convulsions and unable to feed at all. Whereas the signs of clinical severe infection include fast breathing (60 breaths per minute or more, severe chest in-drawing, high body temperature or fever (37.5^o^C or above) or hypothermia (<35.5^o^C), movement only when stimulated and not feeding well.

Page 26, line 577.*Panel 1.Definitions and management strategies of young infants with signs of PSBI – adapted by Government of Ethiopia from WHO Guideline [14]*

**Comment #16**

Fast breathing pneumonia is not a typical term. Do you mean respiratory distress that would indicate a possible pneumonia? How was this identified?

**Authors’ response**

Thanks. Based on the WHO PSBI classification [reference 14], the fast breathing pneumonia is includes defined as fast breathing (60 breaths per minute or more) as the only sign of PSBI in a young infant 7 – 59 days old.

Page 26, line 577.*Panel 1.Definitions and management strategies of young infants with signs of PSBI – adapted by Government of Ethiopia from WHO Guideline [14]*

**Comment #17**

Were there PDSA cycles? Changes made to the interventions/implementation based on real-time data? The description of all stakeholders and educational interventions as well as process changes were thoroughly described, but a timeline of implementation would be helpful to understand what order changes occurred in (or if it was all at once), and what impact this had on the data.

**Authors’ response**

Thanks. Yes, there were plan-do-study-act (PDSA) cycles. For example, a pregnancy cohort was introduced after we initiated the intervention to track pregnancies and new births. Health center staffs were trained to address the back referral from the health centers to the health posts. As a result, back referrals stopped and pregnancy and new birth tracking got improved.

Page 33; line 593: Table 4. Implementation challenges encountered and solutions provided**.**

**Results**

**Comment #18**

*We estimated that 25% of live births were not identified by HEWs in this study (n=1964)* – why are some not identified and where is this number coming from?

**Authors’ response**

Thanks. The total reported population of the two districts under study was 260884 [reference 16]. The expected births using the regional conversion formula for live births is 3.1%, which gives a rough estimate of 8087 live births in a year. We identified only 7857 births during the course of the study, which was more than one year. We didn’t identify all live births, especially in the beginning of implementation of this research. Thus, we assumed that probably 25% of live births were not identified. This proportion of missing births was also reported from a similar study from Nigeria [22].

Page 13; line 280 – 282. We estimated that 25% of live births were not identified by HEWs in this study, particularly in the early part of the implementation (n=1964), similar to that reported in a similar study from Zaria, Nigeria [22],

**Comment #19**

*In the initial stages of implementation, the rate of identification and management of sick young infants with PSBI by HEWs was low. This improved after six months of implementation (Figure 1).* – the rate is not on this figure, it is the total number of cases, so this is not consistent data reporting.

**Authors’ response**

Thanks. As per the comments of the reviewer, results are presented in the form of proportions than cases.

Figure 1. Proportion of new cases of PSBI identified by HEWs and nurse supervisor in each quarter of study period, January 2016 to August 2017(n= 521)

**Comment #20**

Figure 1 is a bar graph – for quality improvement/implementation research, analysis should be done using a run chart or statistical process control chart (preferred). This would allow statistical significance to be identified. The key interventions contributing to any changes on the chart can be indicated so that correlation can be seen. Data can be broken down into weekly or biweekly so that there are enough data points to make a statistical process control chart.

**Authors’ response**

Thanks. As per the comments from the reviewer, the bar graph is changed to a run graph, a line graph of data plotted over time.

Figure 1. Proportion of new cases of PSBI identified by HEWs and nurse supervisor in each quarter of study period, January 2016 to August 2017(n= 521)

**Comment #21**

What is the baseline data pre-implementation? There is no comparison, only description of results after implementation.

**Authors’ response**

Thanks. We didn’t collect pre-implementation baseline data as these services were not available in the study communities. However, a cluster randomized controlled trial had reported 50% coverage in the ‘intervention area’ of treatment after implementation of a similar PSBI intervention through HEWs when referral was not possible compared to the control area in a population of 640,000 in 3 zones - East Shoa and West Arsi in Oromia Region and Sidama in Southern Nations, Nationalities, and People’s Region (SNNPR)—that are demographically and socioeconomically similar to Ethiopia’s agrarian regions where 85% of the country’s people live [reference 15].

**Deaths**

Comment #22

deaths *occurred among the 144who were referred by HEWs*– typo

**Authors’ response**

Thanks. We have corrected the typo.

**Implementation challenges and solutions**

**Comment #23**

This section should be incorporated into the description of the interventions, not after the results. Preceding barriers can be described first in the context of why the interventions were chosen. If applicable, each problem and solution should be grouped with the description of the intervention itself. Ongoing challenges can be described in the discussion in the context of next steps. It seems much of what is described in this section are the initial barriers identified – this needs to be described earlier in the manuscript as context for the implementation phases.

**Authors’ response**

Thanks. We believe that the appropriate place for the implementation challenges and solution is the result section, as we faced these while implementing this research and not before that.

Page 15; line 319. Implementation challenges and solutions

**Comment #24**

*This resulted in improvement in the skills to carry out activities as per standard case management in the subsequent quarters* – where is this data?

**Authors’ response**

Thanks. We have mentioned that in Table 4.

Page 33; line 593: Table 4. Implementation challenges encountered and solutions provided**.**

**Comment#25**

The discussion of poorly equipped neonatal intensive care units does not seem relevant to the study.

The TSU had been focusing on referral and this has improved significantly after the first quarter. - Where is the data on referral rates? There is no description of this analysis or figure to demonstrate statistical significance. This cannot be stated unless clearly demonstrated.

**Authors’ response**

Thanks. The comment from the reviewer is well taken and the paper is modified accordingly.

**Comment #26**

The discussion needs to relate back to your primary outcomes. Did you achieve your goals?

**Authors’ response**

Thanks. Yes, we believethat we achieved our goals. We identified and treated 87% of the PSBI cases, so the coverage was above 80% listed in our objective. Almost all (98%) who were treated on an outpatient basis received appropriate treatment.

Page 14; lines 283 – 284. Coverage of treatment of PSBI cases in our study area was 87% (854/982).

Page 14; line 296 – 299. All except one (63, 98%) who were treated at health post received appropriate treatment. One was lost to follow-up, but no death was reported.Adherence to treatment was 100% for injection gentamicin and 98% for oral amoxicillin respectively (Table 3)**.**

**Comment #27**

Data is being compared against different studies and conclusions are being made without data analysis showing statistical significance with the implementation of these interventions

**Authors’ response**

We did not collect any baseline data because this intervention was not available before this study. Even, when compared with the previously published data from Ethiopia of 50% coverage [reference 15], our data showed an 87% coverage of treatment for infants with PSBI signs. However, we didn’t perform any statistical analysis.

In line with the reviewer’s comments, in the discussion section we have now compared our data with results from several PSBI implementation research studies that evaluated the feasibility of WHO PSBI guideline implementation at program level in several countries, which have been published since the submission of the paper.

*When we compare our data with other PSBI implementation research studies in various countries, we find some similarities and some differences. Our treatment coverage of 87% was a bit lower than that reported by the study in Zaria, Nigeria (96%) [22]; but was higher than those reported from Malawi (64%) [26]; Lucknow, India (53%) [27] and Kushtia, Bangladesh (31%) [28]. Like our study, high treatment completion rates were also reported by Malawi (95%) [26], Zaria, Nigeria (94.1%) [22] and MaMoni project, Bangladesh (80%) [29] in infants with clinical severe infection treated on an outpatient basis. Our proportion of fast breathing pneumonia in 7-59 day-old infants was 60% among those who were identified at a health post or a health centre, compared to 87% in Kushtia, Bangladesh [28], 30% in MaMoni project, Bangladesh [24], 28% in Malawi [26], 22% in Zaria, Nigeria [22] and 13.3% in Lucknow, India [27].*

Page 16; line 345 – 354

**Comment #28**

Was investigating whether this alternate antibiotic treatment was effective an outcome? There is emphasis on the success of this, but not listed as an objective.

**Authors’ response**

Thanks. It was not our intention to prove the effectiveness of the short course of antibiotics. Evidence proving effectiveness has been demonstrated in the randomized controlled trials [references 10-13], which was used to develop WHO PSBI guideline [references 14] are used of the study. Our main objective was to evaluate the feasibility of WHO PSBI guideline implementation in a Programme setting, as has been explained earlier.

Page 6; line 102 - 105

**Comment*# 29***

*Our* study *had several strengths.* – This should be phrased as keys to successful intervention implementation.

**Authors’ response**

Thanks. We have modified the text as suggested.

Page 19 ; line 408. There *were several keys to successful intervention implementation in our study*

**General issues**

**Comment #30**

Terminology is interchanged and unclear throughout. What is the difference between sick young infants, PSBI, critical illness and clinical severe illness? These terms need to be defined and using distinctly.

**Authors’ response**

Thanks. The comment from the reviewer is addressed in the section on study population. We have developed a panel (see Panel 1) to see the various definitions.

Page 26, line 577.*Panel 1.Definitions and management strategies of young infants with signs of PSBI – adapted by Government of Ethiopia from WHO Guideline [14]*

**Comment # 31**

Unclear whether this study is looking at infants <2 months or 7 – 59 days as both are used.

**Authors’ response**

Thanks. The study wasconducted in 0 – 59 days old young infants. However, young infants who were 7 – 59 days and presented ONLYwith fast breathing were classified as having ‘pneumonia’ and were treated with oral amoxicillin without the need for referral.

See above (panel 1)

**Comment #32**

More sophisticated data analysis should be used. For quality improvement/ implementation research, a run chart or statistical process control chart (preferred) is necessary to demonstrate significant change due to interventions.

**Authors’ response**

Thanks. We have revised the figure 1 as a run chart now, which shows that the number of PSBI cases increased over time. The HEWs were able to identify, assess, classify and treat sick young infants with signs of clinical severe infection who refused referral as evidenced form data given in the tables. We believe in this study to evaluate the feasibility of WHO PSBI guideline implementation in a program setting, no sophisticated analysis is required.

**Comment #33**

There is no statistical significance reported for data analysis. The data is reported without any analysis provided. Overall rates are reported but there is no comparison to base line data or trends over time. This is not standard for implementation research and prevents any conclusions to be made in terms of effectiveness of the interventions.

**Authors’ response**

Thanks. We have provided results of descriptive data analysis. This intervention was new in this community before the initiation as this service was not provided in these communities before the implementation research. Our data showed that high treatment coverage was achieved for young infants with signs of PSBI. If one compares with previously published data from elsewhere in Ethiopia of 50% coverage after intervention [reference 15], our results demonstrate a higher treatment coverage of 87%.

**Comment #34**

There is no comparison of post - implementation data to a baseline data set. Baseline rates reported as “low” but the data are not reported. This prevents any conclusions that the interventions were successful.

**Authors’ response**

Thanks. There was no intervention or PSBI services what so ever in the communities before the implementation of our intervention. It wasa new intervention so we could not formally compare the baseline and post implementation data.However, Figure 1 shows the improvement in identification of PSBI cases and data in Table 2 shows those who were treated.

**Comment #35**

A key driver diagram is essential to a quality improvement/ implementation project. This should be included to demonstrate the issues/barriers and linkage with interventions to achieve aims.

**Authors’ response**

Thanks. We have included a Figure 2to the manuscript that helps readers to understand how births and eventually cases of sick young infants were identified, assessed, classified, referred, treated and followed up.

Figure 2: Schematic representation of the identification, assessment, classification, referral, treatment and follow up of the sick young infants.

**Comment #36**

However, the data analysis is not strong enough to support conclusions of success. This manuscript is more distractive of intervention implementation and barriers/solutions than true quality improvement research.

**Authors’ response**

Thanks. Our objective was to evaluate the feasibility of WHO PSBI guideline implementation to achieve high coverage of treatment through implementation research. Our objective was not quality improvement per se, but we did help improve the quality of care for sick young infants with signs of PSBI, when referral was not feasible. Our results show that overall coverage of treatment was high; almost all of the sick young infants who were treated on an outpatient basis were cured by the intervention; there were no deaths in the infants treated at the community level by the health extension workers; and there were no adverse events as a result of the community based management of PSBI cases. We tested if the new intervention could be implemented at the community level by the low level HEWs.

Page 31; line 586. Table 2.

Page 32; line 590. Table 3.

**Comment #37**

There are so many different facets of this project and elements of data described; it may be helpful to narrow the focus so the key outcomes can be more prominent.

**Authors’ response**

Thanks. We believe that in order to assist Programme managers/policy makers who would want to implement this intervention in a program setting details described in this manuscript will be helpful.

**Comment #38**

The manuscript is very long. Please streamline where appropriate.

**Authors’ response**

Thank you for the suggestion. We have tried to improve the organization of information by moving some information in a table and a panel.

**Comment#39**

Occasional grammatical issues and typos throughout

**Authors’ response**

Thanks. The occasional grammatical issues and typos have been addressed and a native English speaker has reviewed the manuscript for errors.

**Reviewer 3**

**General**

**Comment #-1**

Editing is needed to ensure clarity of writing.

**Authors’ response**

A native English speaker has reviewed the manuscript for errors, which have been corrected.

**Introduction**

**Comment #-2**

On the other hand, at the end of ‘Introduction’ section, the authors stated “objective of the study was to implement a simplified antibiotic regimen (2 days gentamicin injection and 7 days oral amoxicillin) in a program setting and to identify and document the challenges and opportunities for implementation at health post level in two regions in Ethiopia, Tigray and Jimma”Organized, triangulated and sequential presentation of both the quantitative and qualitative findings may serve the purpose of this objective.

**Authors’ response**

Thanks. The paper has been revised to address the reviewer comments and hence Jimma is removed from the paper. The findings in this paper are from the Tigray study. Two sites were selected by WHO but worked independently. We have also clarified theobjective and outcomes to make them more specific. We primarily present quantitative findings but in some places qualitative data is also included, especially during monitoring the progress and supportive supervision of the study. We have tried to improve the presentation of results to make them clearer.

**Objective**

**Comment #-3**

There are inconsistencies in between study objective, methods and presentation of results. Although the authors have mentioned that they have reported only the quantitative data, some of the findings are certainly not from quantitative data. For example, the results presented under ‘implementation challenges and solutions’ and in table 3.

**Authors’ response**

Thanks. Please see our response to comment 2 by the reviewer. Also see responses to comments 1 and 5 by reviewer 1.

**Context and setting**

**Comment #-4**

Under ‘context and setting of the study’ of the Methods section, the authors have mentioned “the primary health care unit (PHCU) at the lowest level consists of 5 health posts, one HC and one primary hospital.-------” Later on the authors have stated “a PHCU feeds in to the secondary level of health care”. These are confusing. Needs to be clear. ‘Specifically, they are expected to conduct postnatal home visits on days 1, 3, 7 and 42 as per the MNCH guideline.’ Who are they? Are they the HEWs or women from WDGs? Please make it clear.

**Authors’ response**

Thanks. The health system structure in Ethiopia is a three-tiered health system structure of primary care (primary health care unit), secondary care (general hospital) and tertiary care (referral hospital). The Primary Health Care Unit (PHCU) at the bottom level consists of health posts that serve 5000, health centers that serve 25000 and a primary hospital which serves 100000 people. The PHCU feeds into the secondary level of health care, i.e. general hospital, which serves a population of one million and the general hospital in turn feeds in to the referral hospital which serves about 5 million people.

Page 6 - 7; line 119 – 125

The HEWs are expected to conduct postnatal home visits on days 1, 3, 7 and 42 as per the Federal Government Maternal newborn and child health (MNCH) guideline^[3]^.

Page 7; line 128 - 129

**Design**

**Comment #-5**

Study design is not clear. Detailed description of the study design is needed for clear understanding.

**Authors’ response**

Thank you for the comment. It was prospective implementation research study design. Data were collected prospectively from each young infant up to 2 months in the study communities from January 2016 to August 2017

Page 7; line 133 - 134.

**Study population -**

**Comment #-6**

First sentence under ‘study population’ of the methods section needs to be rephrased.

**Authors’ response**

Thanks. It is rephrased and modified to address the reviewer comments. All babies born during the study period were included in the study.

Page 7; line 141

**Interventions**

**Comment #-7**

First five sentences under ‘Interventions’ do not provide any information relating to intervention. These should go either under ‘Introduction’ or study setting’. These are relating to Ethiopian health system and already briefly described under ‘Introduction’ section, though.

**Authors’ response**

Thanks. These statements have been removed from the intervention section as per the suggestion from the reviewer.

Page 10; line 19, Intervention

**Ethical consideration**

**Comment #-8**

Under ‘ethical considerations’, the last sentence is not clear. It needs to rephrased for clarity.

**Authors’ response**

Thanks. It is rephrased as per the suggestion from the reviewer.*The study involves repeated visits to the households. So, series of oral informed consents were obtained for participation in the study, i.e., for home visits during pregnancy and child birth; for enrolment when the infant gets sick; for treatment; and for follow up visits.*

Page 13; lines 270 - 273

**Comment #-9**

Data analysis is not clear

**Authors’ response**

Thanks. It is edited as per the suggestion from the reviewer.*Quantitative data were cleaned and entered into SPSS version 20 software. After checking for missing data, outliers and invalid values, descriptive analysis was conducted to report frequencies, coverage and proportion of sick young infants with clinical severe infection, critical illness, pneumonia, local bacterial infections; treatment failures; referrals and treatment outcomes along with relevant information regarding PSBI management and treatment at the community level.*

Page 13; lines 262 - 266

**Conclusion**

**Comment #-10**

Before drawing a conclusion like ‘when referral is not feasible, PSBI management can be implemented safely and effectively at health posts by HEWs in the existing health system in Ethiopia”, the author should perform analysis comparing the cases under different variables, between health posts and health center, and show how statistically significant the results are. For example, compliance to treatment: health posts vs. health centers; declared clinically treatment success: health posts vs. health centers/hospitals

**Authors’ response**

Thanks. We have reported the treatment outcomes of PSBI cases treated at the health posts by the health extension workers based in the community. It wasnot the intention of the paper to compare the treatment outcomes in the various health facilities: health posts, health centers and hospitals, although we have tried to provide some outcome data from those who were treated at the hospital.

**Reference**

**Comment #-11**

The authors should use a reference for making such a statement ‘our data show that management of PSBI when referral is not feasible is possible in the Ethiopian context in line with data reported previously.’ (first sentence of the discussion section).

**Authors’ response**

Thank you for pointing this out. The reference to this statement has been included as ‘reference 15’ - Hailegebriel TD, Mulligan B, Cousins S, Mathewos B, Wall S, Bekele A, et al. Effect on neonatal mortality of newborn infection management at health post when referral is not possible: a cluster randomized trial in rural Ethiopia. Glob Health Sci Prac. 2017; 5(2):202-16.

1. World Health Organization. WHO Recommendations on Postnatal Care of the Mother and Newborn. Geneva: WHO. 2013. Available at <https://apps.who.int/iris/bitstream/handle/10665/97603/9789241506649_eng.pdf?sequence=1>
2. World Health Organization. Integrated management of childhood illness. Management of the sick young infant aged up to 2 months Chart Booklet. World Health Organization, Geneva 2019. <https://apps.who.int/iris/bitstream/handle/10665/326448/9789241516365-eng.pdf?ua=1>
3. Federal Democratic Republic of Ethiopia. Postnatal Care. Blended Learning Module for the Health Extension Programme. Health Education and Training (HEAT) in Africa. Federal Democratic Republic of Ethiopia, Ministry of Health
