# Supplementary material for: Innovative approach for potential scale-up to jump-start simplified management of sick young infants with possible serious bacterial infection when a referral is not feasible: Findings from implementation research
Source: PLoS One. 2021 Feb 5;16(2):e0244192. doi: 10.1371/journal.pone.0244192 (PMC7864440; doi:10.1371/journal.pone.0244192)
Supplement: Attachment — Submitted filename: Point by point responses_reviewers_Oct17102020_CLEAN_SQ.doc [file pone.0244192.s003.doc]

**Point by point responses to reviewers’ comments**

**Journal:** PLOS ONE

**Manuscript ID**: PONE-D-08171

**Manuscript Title*:*** *Innovative approach for potential scale-up to jump-start simplified management of sick young infants with possible serious bacterial infection when a referral is not feasible: findings from implementation research*

**Reviewer 2**

1. **General:**

**Comment # 1**
- review grammar thoroughly, there are scattered missing articles (“the”, “an”) and incomplete sentences.

**Author’s response:**

Thank you. we have corrected the grammatical mistakes and the manuscript has been reviewed by a native English speaker.

**Comment # 2**
-Description of intervention planning and stakeholders is much improved.

**Author’s response:** Thank you.

**Comment # 3**
-Manuscript is generally much easier to understand from an outside perspective.

**Author’s response:** Thank you.

**Comment # 4**

-Were the interventions adapted/adjusted throughout the study period based on the data or was this system implemented at once and then data reviewed at the end of the study period. There are barriers and solutions described but it is not clear if these were determined based on data or subjective reporting of issues/concerns.

**Author’s response:**

Thank you. Since the study was implementation research, the interventions were adapted or adjusted based on the data collected. The sources of the data for the adjustment of interventions were quarterly review meetings, clinical mentoring, monitoring and evaluation checklist completed during the supportive supervision and follow up data collected using the CRFs (Case Reporting Forms).

**Comment # 5**

-You address barriers and solutions and that quality control was monitored monthly, however, the data is only displayed as totals without showing change over time. How often was the data analyzed?

**Author’s response:**

Thank you. We have presented our data in such a way that changes over time are displayed against the target and coverage (Please see Figure 1). The follow-up data from the case report forms were collected every month. The analysis was carried out quarterly. The field supervisor visited the health facilities every month for supportive supervision and clinical mentoring and provided on-site feedbacks to the problems encountered.

**Comment # 6**
-Since there is no baseline data reported, you should explain why this is the case and that you used comparisons of other sites without your interventions. Even changes in the initial quarter to the final quarter could show improvement as the interventions were accepted and modified but there doesn’t seem to be any comparison over time. The treatment coverage rate referenced early in the paper is not restated compared to your results in the discussion to show that you achieved your aims. It is also not clear if this was a similar or the same community.

**Author’s response:**

Thank you. The management of PSBI in young infants was not available in the study communities before the implementation of this research, therefore no comparative baseline data were available for comparison. But, the coverage of young infants with PSBI managed by the health extension workers (excluding those who directly went to a hospital) showed an improvement from 37% in the first quarter to 78% in the last quarter. We compared our findings with another study from Ethiopia referenced in our introduction [ref 15 in the list of references]^[1]^. Also, our findings were in agreement with the AFRINEST study[ref 11 in the list of references]^[2]^. The absence of direct baseline data is included as a limitation of this study as per the suggestion from the reviewer.

Page 13 line 283 – 285 of the revised manuscript

Page 15, line 327 – 328 of the revised manuscript

Fig 1. Of the revised manuscript

**Comment # 7**
-Much better use of tables and figures to elaborate on the information. Data is more completely described.

**Author’s response:** Thank you.

**Comment # 8**
-The manuscript is still quite long – try to find ways to make the wording more concise and eliminate any unnecessary detail.

**Author’s response:**

Thank you. We have tried to make it as concise as possible.

**2. Specific:**

**Comment # 1**

Line 75- An estimated 2.5 million neonatal deaths worldwide and 1 million deaths in Sub Saharan Africa occurred in 2018 contributing to 36% and 47% of under-5 deaths, respectively
-Is the 1 million included in the 2.5 million or additional? Clarify.

**Authors’ response**

Thank you for the clarification. Yes, the one million deaths are included in the 2.5 million neonatal deaths and the sentence was re-written as follows.

In 2018, an estimated 2.5 million neonatal deaths occurred worldwide, of which 1 million were in Sub Saharan Africa contributing to 36% and 47% of under-5 deaths respectively.

Page 5; line 80 - 81 of the revised manuscript.

**Comment #2**

Line 77 - Neonatal infections primarily bacterial in origin, including pneumonia, sepsis, and meningitis
-Sepsis is a clinical diagnosis that can result from a number of types of bacterial infections (including pneumonia and meningitis). Do you mean bacteremia? Clarify

**Authors’ response**

Thank you. Sepsis refers to the presence of bacteria in the blood with clinical manifestations. But, bacteremia is the presence of bacteria in blood. Hence it implies sepsis not bactermia.

Page 5; Line 82 -83 of the revised manuscript.

**Comment #3**

Line 110-113 – too much detail on farming/agriculture that is not applicable to the study.

**Authors’ response**

Thanks. We have removed the redundant information from the revised manuscript. The information was included to address the comments from another reviewer.

**Comment #4**

Line 133 – Previously stated that this is implementation research, however study design here says longitudinal study design.

**Authors’ response**

Thank you. The research design is a longitudinal study design since data were collected prospectively from each young infant through a period of 20 months. But, the strategy used to implement the research design was implementation research. We have revised the text to make it clearer.

Page 7; line 135 of the revised manuscript.

**Comment #5**

Line 143 – incomplete sentence

**Authors’ response**

Thank you for the comment. Addressed as per the reviewer’s comments.

All babies born during the study period were included in the study. Young infants up to 2 months of age with one or more signs of PSBI (Panel 1) who were permanent residents of Raya Alamata and Raya Azebo districts were included.

Page 7 ; line 147

**Comment #6**

Line 145-147 – There seems to be 2 primary outcomes – identification of 80% of infants and treatment of 80% of infants. Please make this statement clearer.
Secondary outcomes – did you have any targets for these?

**Authors’ response**

Thank you for the clarification.

The sentence is rewritten as follows: The primary outcome of this study was identification of at least 80% of the PSBI cases and provision of appropriate treatment for at least 80% of those identified cases of PSBI in young infants aged up to 2 months. We did not have any targets for the secondary outcomes but we monitored their occurrences.

Page 7; line 149 - 151	of the Revised Manuscript.

**Comment #7**

Line 155 - swelling local to injection site
-Local swelling at injection site

**Authors’ response**

Thanks. The sentence was corrected as per the recommendation.

Local swelling at the injection site

Page 8; Line 159- of the revised manuscript.

**Comment #8**

Line 192- woreda is not a commonly known word

**Authors’ response**

Thank you. We have replaced the word woreda by the district.

Pages10; line 196 of the revised manuscript.

**Comment #9**

Line 228-231 – since this was not an issue, consider eliminating this section

**Authors’ response**

Thank you. Supplies and commodities section is removed from the manuscript.

**Comment #10**

Line 247 – CRF is defined later and should be defined with this first acronym use
Authors’ response

Thanks. Comment is well taken and corrected. Case reporting forms (CRFs)

Page 12; line 247

**Comment #11**

Line 255-257 – redundant, information provided earlier and this is not specific to data collection

**Authors’ response**

Thank you for the comment. Redundant information is removed.

**Comment #12**

Line 260 – Earlier you discuss only Tigray, not Jimma.
**Authors’ response**

Thank you. The sentence is removed.

**Comment #13**

Line 265 – I do not believe treatment failure is defined.

**Authors’ response**

Thank you for this comment. Treatment failure was defined as clinical deterioration at any time and/ or failure to respond on day 4 of treatment.

Page 12; line 261 – 262 of the revised manuscript

**Comment #14**

Line 261 – How often was data reviewed/analyzed? Was this done throughout the study period or retrospective on conclusion of the study period?

**Authors’ response**

Thank you. Data was reviewed frequently on arrival to the data management center at Mekelle University for its completeness and was analyzed quarterly for the review meetings. Complete data analysis was done at the end of the project.

**Comment #15**

Line 288 – Was there a goal for the percent identified by HEWs? If so, please add target line to figure. I would write out sentence with your initial percent (8.4%) and final percent (29.2%) here in results in addition to demonstrating in figure.
**Authors’ response**

Thank you.

Yes. The HEWs were expected to identify at least 80% of PSBI cases reported from the districts. We have modified figure 1 to include coverage from the target than the proportion. The coverage of identification of PSBI by the HEWs improved 37% in the first quarter to 78% in the last quarter. This coverage doesn’t include those who went directly to the hospital.

Page 13 line 280 – 281 of the revised manuscript

Page 15, line 323 – 324 of the revised manuscript

Figure 1 in the revised manuscript

.

**Comment #16**

Results – serious adverse effects was listed as a secondary outcome and this is not addressed in results

**Authors’ response**

Thank you for the comment. It is included in the result section. No serious adverse effect or death was reported.

Page 14, line 288

**Comment #17**

Figure 2 – The depiction of this data is confusing. It appears that this is the total number of cases of PSBI identified by the HEWs and how many were identified of this total through each quarter. It does not reflect the number appropriately identified or categorized which is the actual information of interest. I’m not sure you can interpret that a better identification rate occurred because there may have just been more cases during a particular quarter. Your denominator to say identification increased would have to include infants with delayed identification or identification by other means, showing how many the HEWs identified vs missed. You could instead show improvement in their skills based on the number per quarter that were misclassified based on review by the pediatrician, with this number hopefully decreasing as the intervention progressed.

**Authors’ response**

Thank you. Figure 2 was included after a suggestion from reviewer 1 and has been accepted favourably. This figure intended to show the flow from livebirths to PSBI cases being identified and managed at different places of health care. We also documented their outcomes. We have revised figure 1 to provide information about the number of PSBI cases identified in each quarter.

**Comment #18**

Line 331 – You discuss the referral acceptance rate, however, this was not included in your primary or secondary outcomes

**Authors’ response**

Thank you for the comment. It is included as a secondary outcome.

Page 8; line 156 of the revised manuscript.

**Comment #19**

Line 360-361 – incomplete sentence

**Authors’ response**

Thank you for the comment. The incomplete sentence was removed.

**Comment # 20**

Line 428 – this is very subjective.

**Authors response.**

Thank you. This statement emanated from the findings presented in Figure 1. The coverage of PSBI identification during the early stages of the implementation was 30% and subsequently increased up to 62% due to the training provided to the HEWs and WDG leaders (Table 4).

**Comment # 21**

Implementation challenges and solutions should be part of the methods section. This is an essential part of your interventions and the description of the implementation process.

**Author’s response**

Thank you for your comment. We, however, believe that the implementation challenges and solutions were a result of our implementation research and we couldn’t have anticipated these challenges and their solutions beforehand.

**Reviewer 3**

Reviewer #3: Thanks to the authors. However, still there are inconsistencies in between study objective, methods and presentation of results. The authors mentioned that the objective of this implementation research was to implement a simplified antibiotic regimen (2 days gentamicin injection and 7 days oral amoxicillin) for management of sick young infants with PSBI in a programme setting when referral was not feasible to achieve an overall treatment coverage of at least 80% in two districts in Tigray, Ethiopia (line 102-105). The objective of an implementation research is to produce evidence, not to implement a program only.

Without having any counterfactuals and based only on descriptive statistical analysis, it is not sufficient to draw a conclusion that when referral is not feasible, young infants with PSBI can be managed appropriately at health posts by HEWs in the existing health system in Ethiopia. Rather the authors could explore the factors associated with the high coverage (identification and treatment of 87% young infants with PSBI) with inferential analysis, and with the support of more qualitative data.

The authors also mentioned that they collected both the quantitative and qualitative data and they also reported some qualitative findings. It was not mentioned in the method section how the qualitative data were collected and analysed.

**Author’s responses**

- Inconsistencies – In light of the comments, we have made the objective, methods and result sections consistent. The objectives are made to describe concisely what the research is trying to achieve (the results) through the approaches described in the method section. Implementation research in health is essential to improving the understanding of the challenges health systems face and how they impact implementation. Thus, in this implementation research, we have identified the contextual challenges affecting the implementation of PSBI in a program setting in two districts from Tigray, Ethiopia.

Thus the objective of this study was to implement a simplified antibiotic regimen (2 days gentamicin injection and 7 days oral amoxicillin) for management of sick young infants with PSBI in a programme setting when a referral was not feasible to identify at least 80% of PSBI cases, achieve an overall adequate treatment coverage of at least 80% and document the challenges and opportunities for implementation at the community level in two districts in Tigray, Ethiopia.

Page 6; line 105 – 110 of the revised manuscript

- Data analysis – We used descriptive analysis for analysis. This intervention was new in this community and before the initiation of this implementation research, this service was not provided in these communities. Our data showed that high treatment coverage was achieved for young infants with signs of PSBI. If one compares with previously published data from elsewhere in Ethiopia of 50% coverage after the intervention [reference 15 cited as footnote 1 on page 2], our results demonstrate a higher treatment coverage of 87%.
- Methods for qualitative data – Both quantitative and qualitative data were collected. But, in this manuscript, we primarily reported the quantitative findings. However, the results summarized in table 4 were qualitative data synthesized from the case report forms, review meetings, clinical mentoring visits and supportive supervision reports.

1. Hailegebriel TD, Mulligan B, Cousins S, Mathewos B, Wall S, Bekele A, et al. Effect on neonatal mortality of newborn infection management at health post when referral is not possible: a cluster randomized trial in rural Ethiopia. Glob Health Sci Prac. 2017; 5:202-16.
2. African Neonatal Sepsis Trial (AFRINEST) Group, Tshefu A, Lokangaka A, Ngaima S, Engmann C, Esamai F, et al. Simplified antibiotic regimens compared with injectable procaine benzylpenicillin plus gentamicin for treatment of neonates and young infants with clinical signs of possible serious bacterial infection when referral is not possible: a randomised, open-label, equivalence trial. Lancet. 2015; 385:1767–76.
